# Deep Learning on Histopathological Images Enables Accurate Diagnosis of Common B-Cell and Hodgkin Lymphoma

**DOI:** 10.3390/cancers18193237

**Published:** 2026-10-08

**Authors:** Peisen Zhang, Changjiang Yan, Meilin Sun, Shaojie Ding, Lingmei Li, Wenfeng Cao, Lu Cao

**Affiliations:** 1 Tianjin Key Laboratory of Integrated Design and Online Monitoring for Light Industry & Food Machinery and Equipment, Tianjin University of Science and Technology, Tianjin 300222, China; zpstust@tust.edu.cn (P.Z.); cj@mail.tust.edu.cn (C.Y.); 2 Department of Pathology, Tianjin Medical University Cancer Institute & Hospital, National Clinical Research Center for Cancer, Tianjin’s Clinical Research Center for Cancer, Key Laboratory of Cancer Prevention and Therapy, Tianjin 300060, China

**Keywords:** lymphoma diagnostic system, whole slide image, artificial intelligence, deep learning

## Abstract

Accurate subtyping of lymphomas is critical for guiding treatment decisions and predicting patient outcomes, yet it remains challenging due to overlapping morphological features. This study aimed to develop and validate a deep learning-based diagnostic system, designated BH-LymphDS, to accurately differentiate the three most prevalent lymphoma subtypes: Diffuse Large B-cell Lymphoma (DLBCL), Follicular Lymphoma (FL), and Hodgkin Lymphoma (HL). The system integrates a Swin Transformer backbone with a clustering-constrained attention multiple instance learning (CLAM) algorithm and a k-nearest neighbors (KNN) classifier, supporting weakly supervised learning directly from whole-slide images without manual region-level annotation. A total of 1910 whole-slide images (WSIs) from diagnostic biopsies were utilized for model development—the largest cohort to date in this field. The performance of nine distinct deep learning architectures was systematically compared. Model efficacy was rigorously evaluated through five-fold cross-validation and further validated on independent external datasets from TCIA and TCGA. Additionally, a comparative analysis was conducted against the diagnostic performance of three experienced hematopathologists. In the internal testing cohort, BH-LymphDS achieved a micro-average AUC of 0.955 and an accuracy of 92.5%. During cross-validation, the system attained a superior micro-average F1-score (0.871) compared to the average of the three hematopathologists (0.851). The Swin Transformer emerged as the optimal backbone, and gradient-weighted class activation mapping (GradCAM) confirmed that the model’s decisions were based on legitimate morphological features such as nuclear atypia and cellular growth patterns. Our findings demonstrate that an advanced AI system can satisfy the rigorous requirements for accurate lymphoma subclassification on histopathological slides. The BH-LymphDS shows strong potential as a reliable auxiliary diagnostic tool to enhance clinical workflow efficiency and diagnostic consistency.

## Simple Summary

Lymphoma, a complex cancer of the lymphatic system, includes several subtypes that require different treatments. However, distinguishing these subtypes under a microscope is notoriously difficult, often requiring expensive and time-consuming additional tests. To address this challenge, we developed an artificial intelligence (AI) system named BH-LymphDS, which functions as a digital pathology assistant to analyze routine biopsy slides. By training our system on the largest known collection of lymphoma images (over 1900 slides), we found that it could identify the three most common lymphoma subtypes—Diffuse Large B-cell Lymphoma, Follicular Lymphoma, and Hodgkin Lymphoma—with an accuracy matching or even surpassing that of three experienced hematopathologists. Our findings suggest that this AI tool could help pathologists make faster, more accurate preliminary diagnoses, potentially reducing the need for certain supplementary tests and improving diagnostic efficiency, particularly in settings with limited specialist resources.

## 1. Introduction

Lymphoma, a complex and diverse group of diseases, has multiple subtypes, and among them, Diffuse Large B-cell Lymphoma (DLBCL), Follicular Lymphoma (FL) and Hodgkin Lymphoma (HL) are the three most frequent subtypes, accounting for approximately 55–75% of all lymphomas [1,2,3,4,5,6]. Lymphomas are heterogeneous lymphoid malignancies with diverse morphological, immunophenotypic, and clinical features. DLBCL is an aggressive mature B-cell neoplasm characterized by diffuse proliferation of large neoplastic B cells. FL is typically an indolent germinal-center B-cell lymphoma with a follicular growth pattern and frequent BCL2 rearrangement. HL is characterized by Hodgkin/Reed–Sternberg (HRS) cells within an inflammatory background, and classic HL usually shows CD30 and CD15 expression. Although IHC and molecular tests are essential for final diagnosis, H&E morphology remains the first and most widely available diagnostic step. The fact that various subtypes require different therapies, represent different responses to treatments and are generally related to different prognoses makes the accurate classification of lymphoma subtype crucial.

Accurate microscopic diagnosis of lymphoma subtypes remains challenging, even for experienced hematopathologists. In the process of diagnosing lymphoma, after carefully observing the hematoxylin and eosin (H&E)-stained tissues, pathologists need to analyze the morphological characteristics of the cells within the tissues, and determine one or a few most probable preliminary diagnoses. These preliminary diagnoses represent only the first stage in a lengthy and meticulous process. Subsequently, they still need to conduct further verification through various advanced methods, such as immunohistochemistry (IHC) stains, flow cytometry or genetic testing, which require specialized personnel, expensive equipment and reagents. Obviously, this process heavily relies on the preliminary diagnosis results made by pathologists based on their experience. Erroneous preliminary diagnoses naturally lead to multiple verification procedures, thus prolonging the diagnosis turnaround and increasing healthcare costs. However, not all pathologists can rely on extensive hematopathological experience, so experienced hematopathologists are valuable and in short supply because of the requirement for specialized training and long-term practice.

Currently, artificial intelligence (AI) based on whole slide imaging (WSI) has shown great potential in pathological classification. WSI refers to digitizing conventional H&E-stained slides to generate corresponding digital pathology images. AI has the capability to reduce the inter and intra observer variability by objective analysis and detect high dimensional features which cannot be recognized by human beings. With advancements in WSI technology and the Food & Drug Administration approval for using a digital pathology system in primary diagnosis, AI has been actively studied across all areas of pathology [7]. Recent systematic reviews have further highlighted the potential of AI in DLBCL diagnosis [8,9]. Using AI as an auxiliary tool can assist pathologists in classifying lymphoma subtypes more accurately and efficiently, reducing the number of IHC-stained sections required for accurate diagnosis. This is especially important for regions with limited medical resources and for general pathologists who lack experience in diagnosing lymphoma. We hypothesized that a weakly supervised deep learning pipeline combining a hierarchical Swin Transformer, attention-based multi-branch CLAM, and a KNN classifier could accurately differentiate DLBCL, FL, and HL on routine H&E-stained WSIs, with performance comparable or superior to experienced hematopathologists, and could generalize to independent external WSI cohorts.

In this study, we developed a B-cell and Hodgkin Lymphoma diagnostic system (BH-LymphDS) to distinguish DLBCL, FL and HL and compared the results with the diagnoses of experienced hematopathologists. The proposed BH-LymphDS demonstrated excellent performance in both five-fold cross-validation and the external testing sets constituted by lymphoma slides from The Cancer Imaging Archive (TCIA) and The Cancer Genome Atlas (TCGA) database [10].

## 2. Materials and Methods

### 2.1. Data Preparation and Processing

A total of 1910 H&E-stained slides from 1517 lymphoma patients, who underwent a biopsy diagnosis between 2018 and 2022 at Tianjin Cancer Hospital (TCH), were scanned by KFBIO PRO400 (Ningbo Konfoong Bioinformation Company, Ningbo, China) at a resolution of 40×. The average age of these patients is 55.9 ± 14.9. These 1910 biopsy specimens were obtained from various anatomical sites (Supplementary Table S3) and comprised 838 DLBCL cases, 869 FL cases, and 203 HL cases. The lymphoma subtype of each specimen was confirmed by immunohistochemistry (IHC) or genetic testing, with detailed inclusion criteria for each subtype described below:

(1) Diffuse large B-cell lymphoma (DLBCL): All enrolled cases were diffuse large B-cell lymphoma, not otherwise specified (DLBCL, NOS). Each case was verified to derive from mature B cells via immunohistochemistry (CD19^+^, CD20^+^, CD79a^+^ and/or PAX5^+^), and all tested negative for Epstein–Barr virus encoded RNA (EBER) by in situ hybridization (ISH). High-grade B-cell lymphomas (HGBL) carrying MYC, BCL2 and/or BCL6 rearrangements were excluded from this cohort.
(2) Follicular lymphoma (FL): All follicular lymphoma cases were diagnosed by combined morphological observation and immunohistochemical staining (CD20^+^, CD19^+^, CD79a^+^, PAX5^+^, CD10^+^, BCL6^+^, BCL2^+^).
(3) Hodgkin lymphoma (HL): This category includes classic Hodgkin lymphoma (cHL). The diagnosis of classic Hodgkin lymphoma relies on the identification of Hodgkin/Reed–Sternberg (HRS) cells with CD30^+^ and CD15^+^ expression within an inflammatory background.

The dataset was split at the patient level. All WSIs from the same patient were assigned exclusively to either the training cohort or the internal testing cohort, ensuring no patient overlap between training and testing.

The TCH cohort consisted of eligible pretreatment diagnostic biopsy specimens obtained between 2018 and 2022. Inclusion required sufficient H&E-stained tissue for WSI scanning and a confirmed diagnosis by IHC and/or genetic testing. Cases with poor tissue quality, missing H&E slides, or HGBL with MYC/BCL2/BCL6 rearrangements were excluded. Of the included WSIs, 1449 (75.9%) were core needle biopsies and 461 (24.1%) were forceps/endoscopic biopsies. Anatomical distributions are summarized in Supplementary Table S3. External validation used 203 DLBCL WSIs from TCIA and 26 DLBCL WSIs from TCGA, both derived from surgical resection specimens.

Three hematopathologists independently diagnosed these slides in the TCH dataset, under the circumstance that they already knew that the subtype of the lymphoma had to be one of DLBCL, FL, HL. The H&E-stained lymphoma slides in the TCIA database and the TCGA database were used as independent external testing cohorts, abbreviated as TCIA-DLBCL and TCGA-DLBC respectively. Both datasets consist entirely of surgical resection specimens obtained at initial diagnosis. To compare the classification capabilities of various backbone models, the WSIs of the three lymphoma subtypes within the TCH dataset were equally partitioned into five subsets and then divided into a training cohort and an internal testing cohort at a ratio of 4:1. After selecting the Swin Transformer as the backbone model, the five-fold cross-validation method was used to evaluate the system performance, with the TCIA-DLBCL and TCGA-DLBC datasets serving as external testing cohorts during each cross-validation cycle. This study was approved by the Ethics Committee of Tianjin Cancer Hospital (No. bc20250352, approved in March 2025). All research procedures followed the Declaration of Helsinki. Slides and clinical data were fully anonymized and de-identified before use, with no personal patient information involved. Patient consent was not required due to complete data anonymization.

To address the challenge of handling large-scale digital images such as WSI, a systematic pre-processing strategy was implemented. First, the WSIs were divided into 512 × 512 patches using a non-overlapping partitioning approach, strictly maintaining a resolution of 0.25 μm/pixel. Then, to ensure high-quality data, the background images were removed according to the pixel threshold and bright threshold. In addition, the Vahadane method was used to normalize the color of these patches. During the training process, data augmentations were applied, including random flipping and erasing, and for the testing process, only color normalization was used.

### 2.2. Deep Learning Model Training

Nine different DL models were trained to compare their abilities in classifying lymphoma subtypes. This process was carried out on the OpenMMLab platform [11], and the code with the toolbox MMPreTrain (version 1.2.0) was implemented to ensure the effectiveness of our model training. Transfer learning was implemented to enhance the generalization ability of the models, and the initialized model weights, which had been pre-trained on public datasets, were downloaded from the official repository. Hyperparameters of these backbone models were shown in Supplementary Table S2. After initialization, we continued to train the models using the histopathological images from the training cohort. Subsequently, the trained models were employed for patch level label prediction, the extraction of features from patch images and the generation of GradCAM maps.

### 2.3. Multi-Instance Learning Process

The multi-branch CLAM [12] was used to cluster the patches of each slide to obtain WSI level features. CLAM represents a specific type of MIL-based approach that combines attention mechanisms to identify diagnostically valuable sub-regions (image patches) and instance-level clustering to optimize the feature space, enabling efficient extraction of features from patches for accurate WSI classification. Multi-branch CLAM is an advanced version of CLAM that incorporates multiple branches, each processing image data in different ways such as focusing on distinct feature aspects, and then integrates the outputs of these branches through a specific fusion strategy to comprehensively capture more diverse information and improve the quality of WSI level feature generation for better classification performance. Through this method, the initially dispersed patch-level predictions were effectively amalgamated, producing WSI-level features.

### 2.4. Signature Building

After obtaining the WSI-level features, we filtered these features in order to remove redundant information. For feature selection, initially, all feature lines were standardized with the z-score standardization method. Next, Pearson’s rank correlation coefficient was employed to compute the correlation among features. When there is a monotonically increasing or monotonically decreasing relationship between two features, this coefficient can quantify the closeness of such a relationship. Among the features, if the correlation coefficient between any two features was greater than 0.9, only one of these features was retained. Finally, the least absolute shrinkage and selection operator (LASSO) regression was used for feature selection, since it can shrink the coefficients of some unimportant features to zero, thereby achieving the purpose of variable selection and reducing the risk of model overfitting.

### 2.5. Model Evaluation

After signature building, the KNN method with kd_tree algorithm and uniform weights was selected to develop classifier. The micro-average F1 score was employed as the performance metric, along with accuracy, AUC value, precision, sensitivity and specificity calculations. In addition, GradCAM images were employed to visualize the outcomes of patch-level model training, while t-SNE images were utilized to illustrate the clustering efficacy of WSI-level features.

## 3. Results

### 3.1. Data Acquisition and System Overview

**Figure 1:**
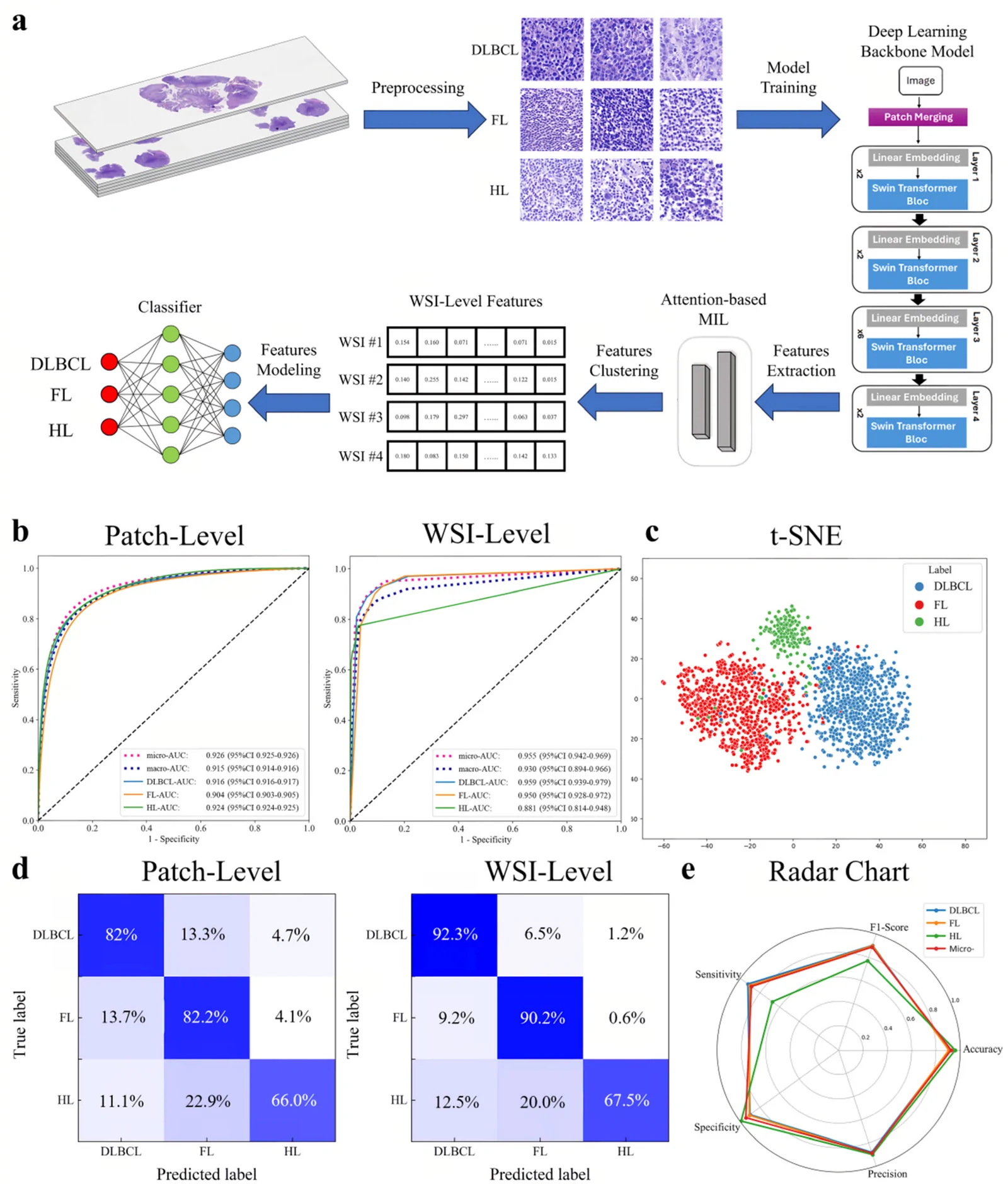
System overview. (**a**) Workflow of the system. Patch-level features were extracted using a deep learning backbone based on Swin Transformer. Then, WSI-level features were clustered using the multi-branch CLAM method. Finally, diagnosis result of each WSI was predicted using a KNN classifier. In the classifier diagram in (**a**), red circles represent the input class labels (DLBCL, FL, and HL), green circles represent hidden-layer neurons, and blue circles represent output nodes. The symbol “#” in “WSI #1–#4” is used as a number sign to indicate the sequence number of whole-slide images, with no additional special meaning. In the classifier module, red nodes represent the three input lymphoma subtype labels, and green nodes represent the intermediate and output nodes of the feature modeling and classification network. (**b**) The ROC curves and AUC values at the patch level and WSI level in the internal testing cohort. The 95% confidence intervals for the AUC values are shown in (**b**). The black diagonal dashed line represents the performance baseline of a random classifier (AUC = 0.5) for reference. At the patch level, the micro-AUC was 0.926 (95% CI 0.925–0.926), macro-AUC 0.915 (95% CI 0.914–0.916), DLBCL-AUC 0.916 (95% CI 0.916–0.917), FL-AUC 0.904 (95% CI 0.903–0.905), and HL-AUC 0.924 (95% CI 0.924–0.925). At the WSI level, the micro-AUC was 0.955 (95% CI 0.942–0.969), macro-AUC 0.930 (95% CI 0.894–0.966), DLBCL-AUC 0.959 (95% CI 0.939–0.979), FL-AUC 0.950 (95% CI 0.928–0.972), and HL-AUC 0.881 (95% CI 0.814–0.948). (**c**) Two-dimensional t-SNE projection of WSI-level features. Blue, red, and green dots correspond to the feature projection points of DLBCL, FL, and HL samples, respectively. Quantitative clustering metrics: silhouette score = 0.4205, Calinski-Harabasz index = 1856.68, Davies-Bouldin index = 0.7419, ARI = 0.7227, NMI = 0.6500, and clustering purity = 0.8759. (**d**) Confusion matrices at the patch level and WSI level. Each cell shows the row-normalized percentage and the absolute number of samples. Rows are normalized according to the true label. The shade of blue in each cell is positively correlated with the row-normalized percentage: a darker color indicates a higher proportion of samples in the true class predicted as the corresponding class. Total sample numbers: patch level, DLBCL *n* = 266,474, FL *n* = 299,748, HL *n* = 77,245; WSI level, DLBCL *n* = 168, FL *n* = 174, HL *n* = 40. (**e**) Radar chart for evaluating system performance at the WSI level in the internal testing cohort. Evaluation metrics were calculated using the One vs. Rest strategy.

The workflow of the BH-LymphDS is shown in Figure 1a. First, the WSIs are preprocessed and cropped into 512 × 512 pixel patches. The BH-LymphDS is trained with a weakly-supervised approach, so each patch image’s label inherits the label of the corresponding WSI. Then, the patches are fed into the deep learning model, which extracts patch-level features during training. The backbone of the BH-LymphDS is constructed based on the Swin Transformer model, which has the ability of multi-scale feature extraction and demonstrated outstanding performance in various computer vision tasks. Subsequently, a multiple instance learning (MIL) method was used to cluster the patch-level features into WSI-level features. The BH-LymphDS employs the multi-branch clustering-constrained attention MIL (CLAM) to extract WSI-level features. The multi-branch attention mechanism in CLAM can extract valuable feature information comprehensively from multiple perspectives. Finally, the BH-LymphDS uses the k-nearest neighbors (KNN) algorithm as the classifier to classify the WSI-level features.

**Figure 2:**
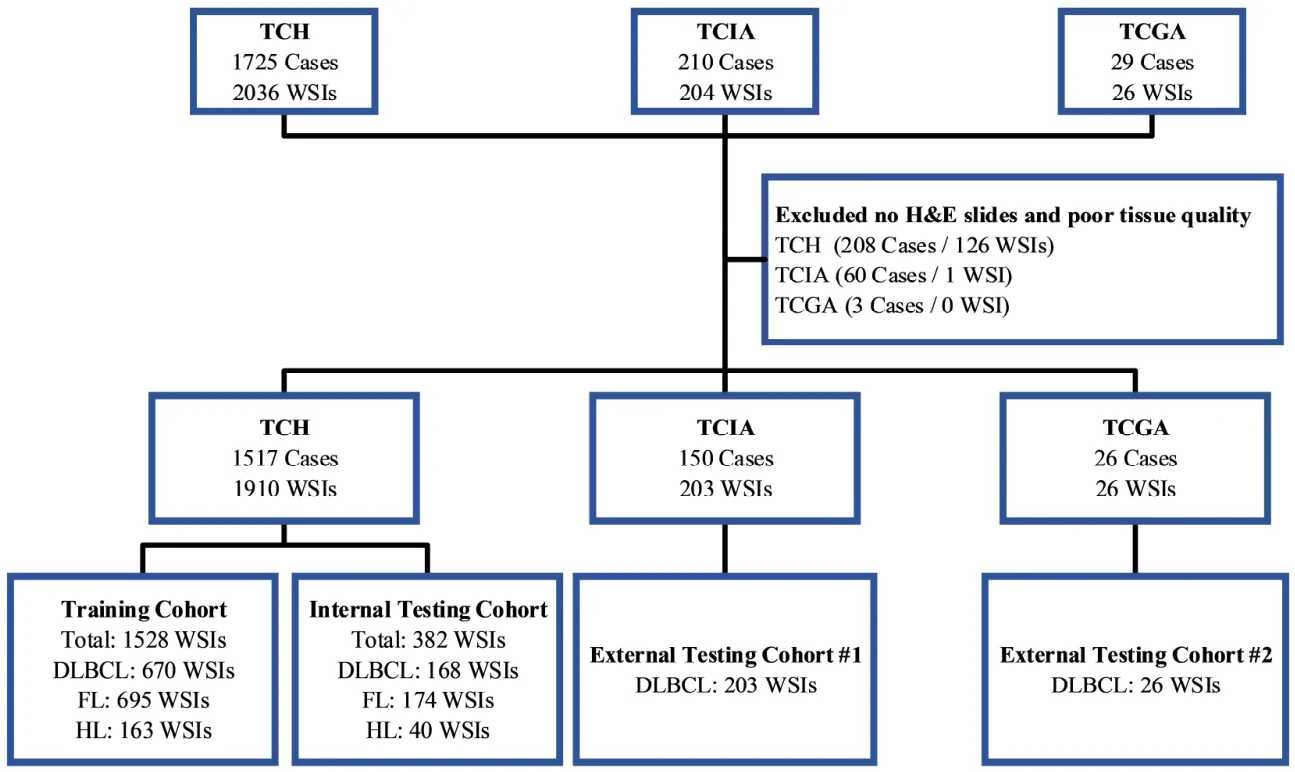
The flowchart for the cohorts used in this research. The lymphoma WSIs from TCH were used as the training cohort and the internal testing cohort in a ratio of 4:1. The lymphoma WSIs from TCIA and TCGA were used as the external testing cohort respectively. The symbol “#” in “External Testing Cohort #1/#2” is used as a standard number sign to indicate the sequential ordinal number of the external validation cohorts, with no additional special meaning.

The flowchart for the cohorts is shown in Figure 2.

A total of 838 DLBCL WSIs, 869 FL WSIs and 203 HL WSIs from Tianjin Cancer Hospital (TCH) were divided into a training cohort and internal testing cohort at a ratio of 4:1. The lymphoma subtypes of all slides have been determined by IHC or genetic testing and are regarded as the ground truth labels. Additionally, 203 DLBCL WSIs from the TCIA database and 26 DLBCL WSIs from the TCGA database were used as the external testing cohort 1 and cohort 2 respectively.

These pretreatment diagnostic biopsies were obtained by either core needle biopsy or forceps/endoscopic biopsy, depending on the anatomical location and accessibility of the lesion. Specifically, 1449 whole-slide images (75.9%) were obtained by core needle biopsy, including superficial and deep lesions accessed by ultrasound/CT-guided percutaneous biopsy. The remaining 461 whole-slide images (24.1%) were obtained by forceps/endoscopic biopsy, primarily from gastrointestinal and other mucosal lesions.

### 3.2. Performance in Internal Testing Cohort

The performance of the proposed lymphoma diagnostic system was evaluated at both the patch-level and WSI-level in the internal testing cohort. The receiver operating characteristic (ROC) curves, along with the area under the ROC curve (AUC), were exhibited in Figure 1b. By comparing the performance at the patch level and the WSI level, it can be concluded that the overall performance of the system as well as its abilities to identify DLBCL and FL have all been improved. We infer that the decrease in the AUC value of HL is due to the fact that its crucial characteristic (Hodgkin cells) shows highly typical manifestations in local patch regions, and there is little variation among these local features across the entire WSI. This inference is consistent with practical pathological experience. For the purpose of evaluating the efficacy of the MIL method in clustering patch-level features into WSI-level features, the t-SNE algorithm was employed to plot the two-dimensional projection of the WSI-level features, as shown in Figure 1c. The discernible differentiation observed among the three groups of DLBCL, FL and HL indicates the effectiveness of the MIL method. The confusion matrices at both the patch level and the WSI level demonstrated the remarkable performance of the proposed system in the classification of DLBCL and FL, as shown in Figure 1d. The evaluation metrics at the WSI level in the internal test cohort are presented in the radar chart of Figure 1e. The micro-average AUC, F1-score and accuracy of the BH-LymphDS were 0.955, 0.887 and 0.925 respectively.

### 3.3. Performance in Cross-Validation and External Testing Cohort

**Figure 3:**
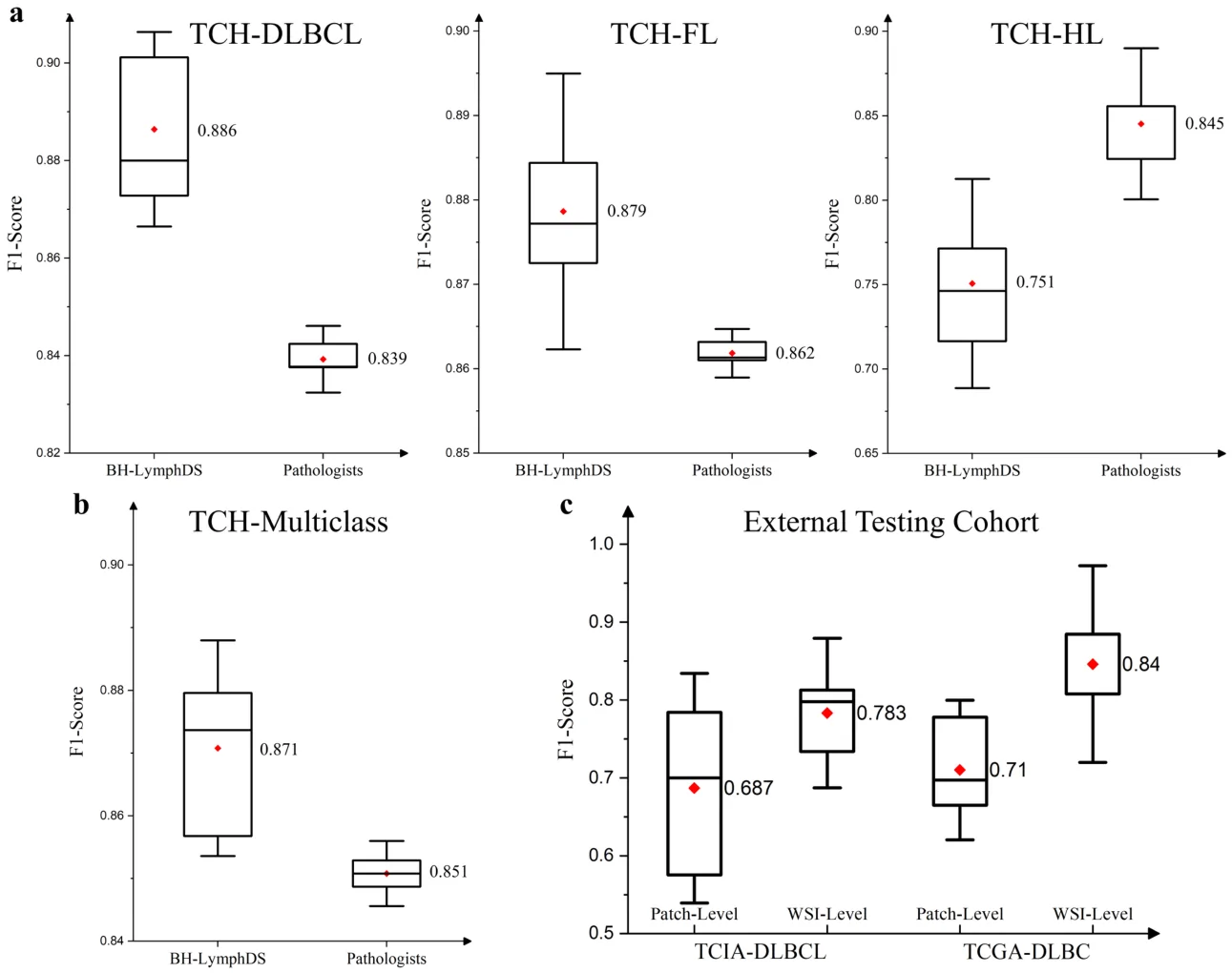
Comparison between the 5-fold cross validation results of BH-LymphDS and the diagnoses of three hematopathologists. Three hematopathologists independently diagnosed all WSIs in the TCH dataset. (**a**) The box plots of the micro-average F1 score were used to compare the efficacy of proposed system and hematopathologists in classifying DLBCL, FL, and HL. (**b**) The box plot of the micro-average F1 score was used to compare the multiclass efficacy of proposed system and hematopathologists in diagnosing these lymphoma subtypes. (**c**) The box plot of the micro-average F1 score was used to illustrate the diagnostic efficacy of proposed system in the external testing cohort.

The TCH dataset was partitioned into five subsets for the implementation of five-fold cross-validation. The deep learning (DL) model was retrained in each cross-validation and all the WSIs of the TCH dataset were once used as the internal testing cohort to evaluate the capability of the system. The deep learning models obtained from each fold of cross-validation training were further evaluated on the external testing cohorts. Five-fold cross-validation performance of BH-LymphDS in the internal TCH cohort and external TCIA/TCGA cohorts, compared with three experienced hematopathologists was shown in Table 1. In addition, three experienced hematopathologists independently diagnosed all the WSIs in the TCH dataset. Before the diagnosis, the three hematopathologists already knew that the subtype of the lymphoma slides should be one of DLBCL, FL, and HL. The cross-validation results present the performance of the proposed BH-LymphDS compared with that of hematopathologists in identifying different types of lymphomas, as shown in Figure 3a. For the identification of DLBCL and FL slides, the BH-LymphDS demonstrated a notable advantage over hematopathologists since it achieved a higher F1-score, which is a crucial metric for assessing the efficacy of classification models. While in the case of HL slides’ identification, the hematopathologists achieved a higher F1 score. When using the micro-average F1 score to compare the multiclass performance, the cross-validation average value of the BH-LymphDS outperformed that of the three hematopathologists (0.871 vs. 0.851), as shown in Figure 3b. The Fleiss Kappa value among the three hematopathologists was 0.823 (*p* < 0.05), indicating substantial inter-observer agreement. The confusion matrices of the diagnoses made by the three hematopathologists were shown in Supplementary Figure S1. When taking the diagnostic results of hematopathologists as the reference standard, which means considering the diagnosis as correct when the model assigned a class that coincides with at least one hematopathologist’s annotation, the precision values for the different classes were as follows: DLBCL 84.5%, FL 93.4%, HL 91.3%. Within both the lymphoma datasets of the TCIA (DLBCL-Morphology) and TCGA (TCGA-DLBC), the average WSI-level F1-score was substantially higher than the corresponding patch-level F1-score, as shown in Figure 3c. In addition, the BH-LymphDS was trained exclusively on diagnostic biopsy specimens obtained before treatment, whereas the TCIA and TCGA datasets consist of surgical resection specimens. This demonstrates that the deep learning model, trained on biopsies, generalizes well to resection specimens from independent sources.

### 3.4. Performance of the System with Various Backbones

**Figure 4:**
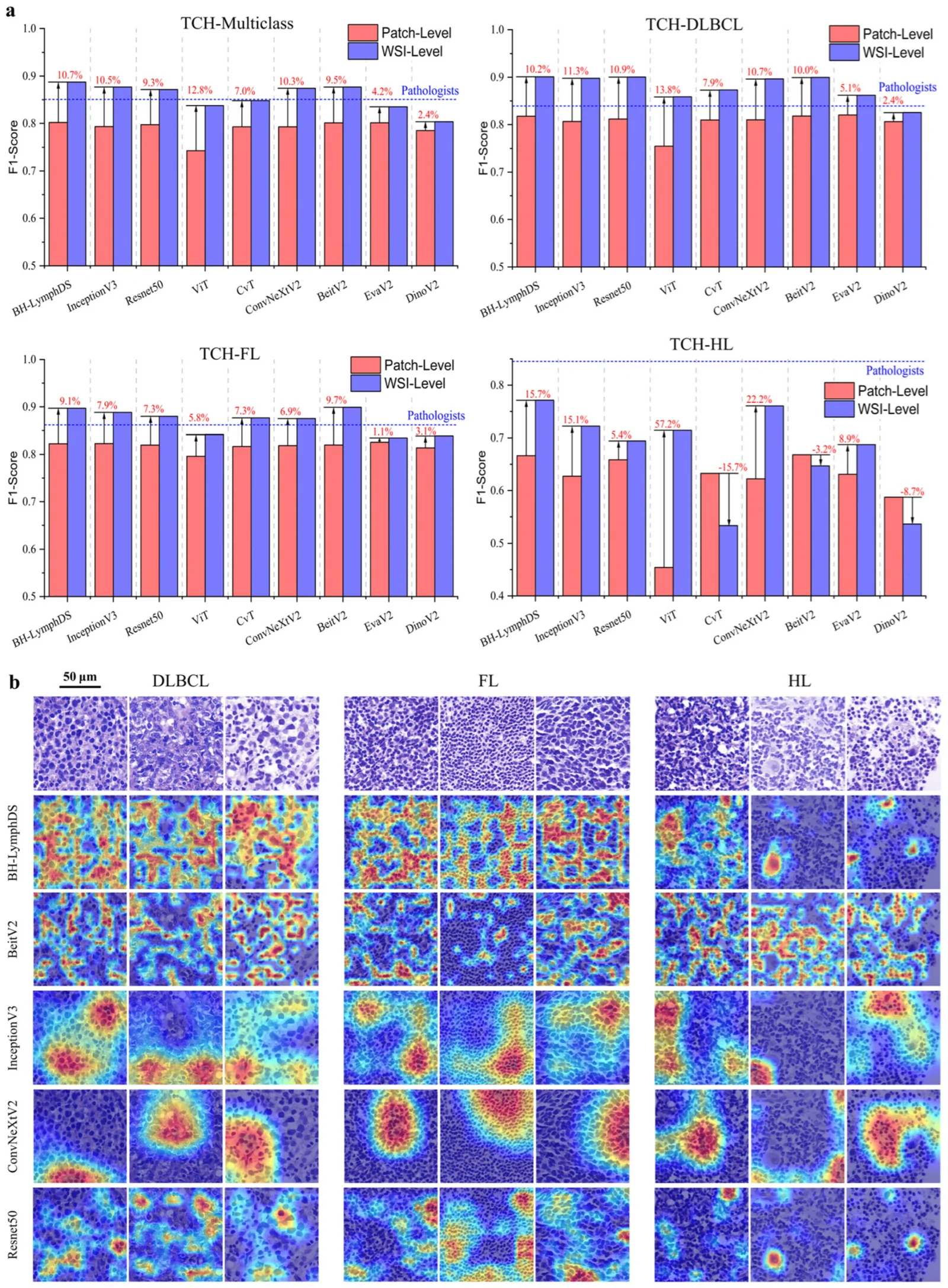
Comparing the performance of various DL backbone models in identifying lymphoma subtypes. (**a**) The micro-average F1 score was employed to contrast the overall multi-classification efficacy of different DL models. Meanwhile, the micro-average F1 scores of these models in distinguishing DLBCL, FL and HL were calculated using the One vs. Rest strategy. The patch level efficacy was presented with a red bar graph, the WSI level efficacy was presented with a blue bar graph, and the blue dotted line indicated the average F1 score of three hematopathologists. (**b**) The top row shows representative H&E-stained patches of DLBCL, FL, and HL, and the remaining rows show GradCAM activation maps of the last convolutional layer for various DL models. Images were acquired at 40× magnification. Scale bar: 50 μm, applicable to all patch images in (**b**).

By replacing the backbone model of the BH-LymphDS, this research compared the multiclass performance of various DL models in classifying lymphoma, as shown in Figure 4a. These DL backbone models included Swin Transformer (BH-LymphDS), Inception_V3, Resnet50, Vision Transformer (ViT), Convolutional vision Transformers (CvT), ConvNext_V2, Beit_V2, Eva_V2 and Dino_V2. The performance of these DL models in classifying DLBCL, FL and HL was also shown in Figure 4a. Compared to other models, the BH-LymphDS, built upon Swin Transformer, presented better efficacy in the multiclass performance as well as in classifying DLBCL, FL, and HL, with a micro-average F1 score of 0.887. The BeitV2 and the InceptionV3 also exhibited good performance in classifying DLBCL, FL, and HL, both with a micro-average F1 score of 0.877. The micro-average F1 scores of ConvNeXtV2 and Resnet50 were 0.874 and 0.872 respectively, but they performed slightly worse in distinguishing HL. The overall multi-classification performance of the BH-LymphDS, InceptionV3, Resnet50, ConvNeXtV2, and BeitV2 was better than that of hematopathologists, while when it comes to identifying HL slides, all nine DL models were inferior to hematopathologists. The detailed evaluation metrics of various backbone models were presented in Supplementary Table S1 and Supplementary Figures S2–S6.

To quantitatively compare the nine backbone models, we summarized their WSI-level micro-average performance in Table 2 (based on Supplementary Table S1). BH-LymphDS achieved the highest micro-average AUC (0.955, 95% CI 0.942–0.969) and the highest micro-average F1-score (0.887), followed by InceptionV3 (AUC 0.935, 95% CI 0.919–0.951; F1 0.877) and BeitV2 (AUC 0.938, 95% CI 0.921–0.954; F1 0.877). The 95% confidence intervals of the different models were compared to aid interpretation. Formal pairwise significance testing among the nine backbone models was not performed because paired WSI-level predictions were not retained for all models; this is stated as a limitation.

To further evaluate whether DL models have learned and extracted correct features, we applied Gradient-weighted Class Activation Mapping (GradCAM) to visualize the activation of the last convolutional layer, as shown in Figure 4b. It highlighted the regions that significantly contributed to the classification, thus providing valuable insights into the decision-making process of the model. In comparison with other models, the proposed BH-LymphDS demonstrated a superior capability in capturing key information. The BH-LymphDS learned the nuclear features that distinguish the three lymphoma subtypes, captured the diffuse growth pattern characteristics of DLBCL and FL, and accurately localized the Hodgkin cells. Therefore, we concluded that the BH-LymphDS identified the respective classes based on cell morphology.

## 4. Discussion

DLBCL, FL, and HL are recognized as the preponderant cancer subtypes within the category of lymphoma. In this research, we have developed a lymphoma diagnostic system, named BH-LymphDS, with the aim of accurately categorizing these three specific and clinically significant lymphoma subtypes. The system integrates a Swin Transformer backbone with the multi-branch CLAM and a KNN classifier. We used over 3 million 512 × 512-pixel patch images cropped from 1910 biopsy tissue WSIs to train models, and up to the present time, this represents the largest data cohort in the field of lymphoma classification. The cross-validation results evinced that the performance of the proposed system surpassed that of professional hematopathologists. Moreover, the system has demonstrated remarkable performance on the external testing cohort, which encompasses the public databases of TCGA and TCIA.

The progression of cancer invariably involves changes in cellular morphology and the microenvironment [13]. Deep learning, with its capacity to extract the characteristics of these changes, exhibits high potential for application within the domain of cancer classification. However, due to the numerous subtypes of lymphoma, no study has yet reported about the development of an AI system with the capacity to achieve a comprehensive and accurate classification of all the fundamental lymphoma subtypes. Shankar et al. developed a machine learning (ML) model with tissue microarray (TMA) slides to classify 8 lymphoma subtypes, and the average accuracy of their model was 0.643 [14]. Hashimoto et al. developed a DL model with lymphoma excision sample slides to classify 6 lymphoma subtypes, and the average accuracy of their model was 0.702 when only using WSI data [15]. Through literature search, Steinbuss et al. indicated that lymphoma subtyping was possible with high accuracy using DL techniques when 2–4 lymphoma subtypes were included for classification [16]. To date, two studies reported models for identifying four lymphoma subtypes [17,18], 11 studies for three subtypes [16,19,20,21,22,23,24,25,26,27,28], and 9 studies for two subtypes [5,17,29,30,31,32,33,34,35,36]. Therefore, we selected the three most common lymphoma subtypes to conduct research.

We used the WSIs of diagnostic biopsy specimens to train the DL models of the BH-LymphDS, considering that the pathological diagnosis obtained from the initial biopsy directly determines the formulation of lymphoma treatment strategies. Meanwhile, it is essential to point out that the proposed system is applicable for the diagnosis of both pretreatment diagnostic biopsy slides and entire tissue specimen slides. Both the TCIA-DLBCL and TCGA-DLBC public datasets are constituted by WSIs scanned from surgical resection specimens, and the BH-LymphDS has achieved satisfactory performance in the cross-validation of these two external testing cohorts, thereby demonstrating its potential efficacy and reliability. Compared with DL models trained using pre-annotated TMAs or patches, the BH-LymphDS can directly diagnose slides without the necessity for pathologists to pre-select cancerous regions in advance, and thus has the potential to serve as an integrated program inside the WSI scanner. It is necessary to state that, instead of manually annotating each region or patch of the WSI, we assigned the label to each WSI, and the cropped patches of each WSI shared the label of the corresponding WSI. This weakly-supervised learning mode has become one of the predominant methodologies within the realm of deep learning model development for histology images since the manual annotation for large-scale datasets is often not practically achievable. However, this mode increases the difficulty in the classification of lymphoma, especially Hodgkin lymphoma. We found that our DL model could concentrate its attention on Hodgkin cells effectively within the patch images, however, after clustering the features of all patches into WSI features using MIL, this localized attention signal was attenuated after multiple-instance learning (MIL) aggregated the features of all patches into a single WSI level representation. This accounts for the relatively weaker ability of the BH-LymphDS to identify Hodgkin lymphoma slides.

In this research, the performance of nine different DL models in classifying lymphoma subtypes was evaluated through cross-validation and public datasets, with the aim of selecting the optimal backbone model. So far, nearly all of the DL models applied in lymphoma classification have been constructed on the basis of convolutional neural network (CNN) architectures. Based on the perusal of published studies, it was found that only Guetarni et al. had constructed a DL model based on the Transformer architecture for the classification of DLBCL subtypes [35]. However, their training cohort only contained 157 patients, which was far less than the patient number of this research. Among the nine deep learning models, Resnet50 and InceptionV3 are classical DL models based on the CNN architecture. Swin Transformer and ViT are classical DL models based on the Transformer architecture. CvT is a DL model combining the architectures of CNN and Transformer. ConvNeXtV2 is a self-supervised DL model based on CNN architecture. BeitV2 and DinoV2 are self-supervised DL models based on Transformer architecture. BeitV2 uses MIM (Masked Image Modeling) for local feature learning while DinoV2 employs contrastive learning for global semantic feature extraction. EvaV2 is a Multi-Modality Algorithm based on the Transformer architecture. The BH-LymphDS selected the Swin Transformer as its backbone model. The Swin Transformer employs the hierarchical sliding window mechanism, which enables the model in maintaining sensitivity to local features and capturing global vision information. This ability to capture multi-scale information can provide more abundant and comprehensive information for cancer classification, and we took it as the principal determinant leading to the model’s optimal performance. As far as we know, we report the first paper to compare the performance of different backbone models in lymphoma subtype multi-classification.

The interpretability of DL models was also evaluated on digital pathology using the GradCAM method [9]. In the research on multi-classification of lymphoma subtypes, this is also the first paper that compared the GradCAM images generated by different DL models. It can be observed that the attention of Swin Transformer is concentrated on the nuclei of the lymphoma subtype. We also observed an interesting phenomenon that the attention areas of Swin Transformer and BeitV2 are almost opposite, but there is no significant difference in their performance at the patch level. As an illustration, for the GradCAM images of HL, Swin Transformer could accurately locate the Hodgkin cells, while the attention of BeitV2 was focused on the surroundings of the Hodgkin cells. In the GradCAM images of HL, the attention positions of Resnet50 are almost identical to those of Swin Transformer. The GradCAM images of InceptionV3 and ConvNextV2 exhibited the typical characteristics of CNN models, with each of them focusing on several key areas. These visualization results proved the interpretability of these DL models.

In the digital pathology field at present, less research has been devoted to lymphoma than to other cancers in digital pathology. The primary reason lies in the fact that lymphoma presents a multiplicity of subtypes and similar morphological characteristics among these subtypes, and therefore, it poses a formidable challenge for AI systems to achieve accurate classification. On the other hand, since the diagnosis of lymphoma relies on experienced hematopathologists who are usually affiliated with comprehensive cancer centers, the imaging data are consequently also concentrated in these hospitals, thereby leading to difficulties in data acquisition. The advantages of our research in contrast to prior published studies are shown as follows: We conducted performance comparisons on different types of deep learning models, thereby enabling us to select the optimal model for constructing the system, and employed the largest dataset of lymphoma biopsy WSI for the training and testing of deep learning models. These enable the BH-LymphDS to achieve satisfactory performance in both cross-validation and public datasets. Our research indicates that an advanced AI system can meet the requirements for accurate classification of lymphoma slides.

## 5. Conclusions

Previous studies have explored DL for lymphoma classification, as comprehensively summarized in the systematic review by Fu et al. [37]. Shankar et al. [14]. developed LymphoML using tissue microarray slides and reported moderate accuracy across eight subtypes. Hashimoto et al. [15]. classified six lymphoma subtypes using excision specimens and achieved an average accuracy of 0.702. Steinbuss et al. [16]. suggested that DL-based lymphoma subtyping is feasible when 2–4 subtypes are included. Guetarni et al. [35]. applied a vision transformer to DLBCL subtyping but with a much smaller cohort. Compared with these studies, our work includes the largest biopsy WSI cohort, systematically compares nine backbones, and validates on independent TCIA and TCGA cohorts.

In this study, we developed BH-LymphDS, a deep learning-based diagnostic system integrating a Swin Transformer backbone with multi-branch CLAM and a KNN classifier to accurately differentiate DLBCL, FL, and HL from H&E-stained histopathological slides. Trained on the largest cohort of lymphoma biopsy WSIs to date (1910 slides from 1517 patients), BH-LymphDS achieved a micro-average AUC of 0.955 and accuracy of 92.5% in internal testing, and outperformed three experienced hematopathologists in five-fold cross-validation (F1: 0.871 vs. 0.851). The system also generalized well to independent external datasets from TCIA and TCGA. Among nine evaluated architectures, the Swin Transformer emerged as the optimal backbone, and GradCAM confirmed that model decisions were grounded in diagnostically relevant morphological features. BH-LymphDS shows strong potential as a reliable auxiliary diagnostic tool to improve efficiency and consistency, particularly in resource-limited settings. Future work will expand coverage to rarer subtypes and improve HL identification.

This study has several limitations. First, only the three most common lymphoma subtypes (DLBCL, FL, and HL) were included, and rare subtypes were not covered. Second, the identification performance for HL was inferior to that of the hematopathologists, and the ability of multi-instance learning to retain rare local features requires further optimization. Third, the training data were derived from a single center, and the external validation cohorts covered only the DLBCL subtype; therefore, further multicenter external validation with a broader range of subtypes is needed. Finally, clinical information and molecular pathological data were not included, and multimodal fusion of histological, clinical, and molecular data may further improve diagnostic performance.

## Figures and Tables

**Table 1:** Five-fold cross-validation performance of BH-LymphDS and comparison with three experienced hematopathologists in the internal TCH cohort, and external validation on TCIA and TCGA cohorts.

Cohort	WSI Counts (Mean ± SD)	Accuracy	Sensitivity	Specificity	F1-Score
TCH-DLBCL	167.8 (±0.4)	0.897 (±0.013)	0.917 (±0.019)	0.882 (±0.013)	0.886 (±0.014)
TCH-FL	173.8 (±0.4)	0.889 (±0.011)	0.883 (±0.011)	0.895 (±0.018)	0.879 (±0.012)
TCH-HL	40.6 (±0.8)	0.956 (±0.008)	0.631 (±0.048)	0.994 (±0.004)	0.751 (±0.045)
TCH-Multiclass	382.2 (±1.3)	0.914 (±0.008)	0.871 (±0.012)	0.935 (±0.006)	0.871 (±0.012)
Pathologist 1	1910	0.901	0.851	0.925	0.851
Pathologist 2	1910	0.899	0.849	0.924	0.849
Pathologist 3	1910	0.902	0.853	0.926	0.852
TCIA-DLBCL	203	0.783 (±0.069)	—	—	0.783 (±0.069)
TCGA-DLBC	26	0.846 (±0.091)	—	—	0.846 (±0.091)

Five-fold cross-validation performance of BH-LymphDS in the internal TCH cohort and external TCIA/TCGA cohorts, compared with three experienced hematopathologists. For BH-LymphDS, values are presented as mean ± SD across five folds. For hematopathologists, values are point estimates from independent diagnosis of all 1910 TCH WSIs. TCH-DLBCL, TCH-FL, and TCH-HL metrics were calculated using the one-vs-rest strategy; TCH-Multiclass metrics are micro-averaged across DLBCL, FL, and HL; External cohorts (TCIA-DLBCL and TCGA-DLBC) contain only DLBCL cases; therefore, sensitivity and specificity are not applicable (—). Ground-truth labels were confirmed by immunohistochemistry and/or genetic testing. Inter-observer agreement among the three hematopathologists was assessed using Fleiss’ kappa (κ = 0.823, *p* < 0.05). DLBCL, diffuse large B-cell lymphoma; FL, follicular lymphoma; HL, Hodgkin lymphoma; TCH, Tianjin Cancer Hospital; TCIA, The Cancer Imaging Archive; TCGA, The Cancer Genome Atlas.

**Table 2:** WSI-level micro-average performance of nine backbone models.

Backbone	Accuracy	AUC (95% CI)	F1-Score
BH-LymphDS (Swin Transformer)	0.925	0.955 (0.942–0.969)	0.887
InceptionV3	0.918	0.935 (0.919–0.951)	0.877
ResNet50	0.914	0.944 (0.929–0.959)	0.872
ViT	0.892	0.939 (0.923–0.954)	0.838
CvT	0.899	0.932 (0.915–0.948)	0.848
ConvNeXtV2	0.916	0.944 (0.929–0.959)	0.874
BeitV2	0.918	0.938 (0.921–0.954)	0.877
EvaV2	0.890	0.930 (0.913–0.946)	0.835
DinoV2	0.869	0.915 (0.897–0.933)	0.804

Values are WSI-level micro-average metrics. AUC values are reported with 95% confidence intervals. Formal pairwise significance testing among the nine backbone models was not performed because paired WSI-level predictions were not retained for all models; this is acknowledged as a limitation.

## Data Availability

The TCH datasets were used with institutional permission through IRB approval for the current study and are thus not publicly available, but are available from the corresponding author on reasonable request. TCGA-DLBC whole-slide data are available from the NIH genomic data commons (http://portal.gdc.cancer.gov (accessed on 20 December 2024)). TCGA-DLBCL-Morphology whole-slide data are available from The Cancer Imaging Archive (https://www.cancerimagingarchive.net/collection/dlbcl-morphology (accessed on 20 December 2024)).

## Supplementary Materials

The following supporting information can be downloaded at: https://www.mdpi.com/article/10.3390/cancers18193237/s1, Figure S1: The confusion matrix of the diagnostic results after three hematopathologists independently diagnosed all WSIs in the TCH dataset. The lymphoma subtypes of all slides have been determined by immunohistochemistry or genetic testing and are regarded as the true labels. Before the diagnosis, the three hematopathologists already knew that the subtype of the lymphoma slides should be one of DLBCL, FL, and HL. Figure S2: The ROC curves and AUC values for predicting lymphoma subtype of patch level images in the internal testing cohort when using various backbone models. Proposed BH-LymphDS takes Swin Transform as the backbone model. Other backbone models include InceptionV3, Resnet50, Vision Transform, Convolutional Vision Transformer, ConvNeXtV2, BeitV2, EvaV2 and DinoV2. Figure S3: The ROC curves and AUC values for predicting lymphoma subtype of each WSI in the internal testing cohort when using various backbone models. Figure S4: The two-dimensional t-SNE projection of the WSI level feature space for predicting the internal testing cohort with the use of various backbone models. Figure S5: The patch level confusion matrix of various backbone models. The normalization was carried out on each row of all the confusion matrices. Figure S6: The WSI level confusion matrix of various backbone model. Table S1: WSI level system performance of various backbone models. Table S2: Hyperparameters of various backbone models. Table S3: Anatomical distribution of WSIs in the TCH cohort.

## Abbreviations

The following abbreviations are used in this manuscript:

AI	Artificial Intelligence
AUC	Area Under the Curve
CLAM	Clustering-Constrained Attention Multiple Instance Learning
CNN	Convolutional Neural Network
DL	Deep Learning
DLBCL	Diffuse Large B-Cell Lymphoma
FL	Follicular Lymphoma
GradCAM	Gradient-Weighted Class Activation Mapping
H&E	Hematoxylin and Eosin
HL	Hodgkin Lymphoma
IHC	Immunohistochemistry
KNN	K-Nearest Neighbors
MIL	Multiple Instance Learning
ROC	Receiver Operating Characteristic
TCH	Tianjin Cancer Hospital
TCGA	The Cancer Genome Atlas
TCIA	The Cancer Imaging Archive
WSI	Whole-Slide Image
