# Supplementary material for: Deep Learning on Histopathological Images Enables Accurate Diagnosis of Common B-Cell and Hodgkin Lymphoma
Source: Cancers (Basel). 2026 Oct 8;18(19):3237. doi: 10.3390/cancers18193237 (PMC13664540; doi:10.3390/cancers18193237)
Supplement: Supplementary file 1 [file cancers-18-03237-s001.zip › cancers-4584493-supplementary.pdf]

Supplementary Information for
Deep Learning on Histopathological Images Enables Accurate Diagnosis of Common B-Cell and Hodgkin Lymphoma

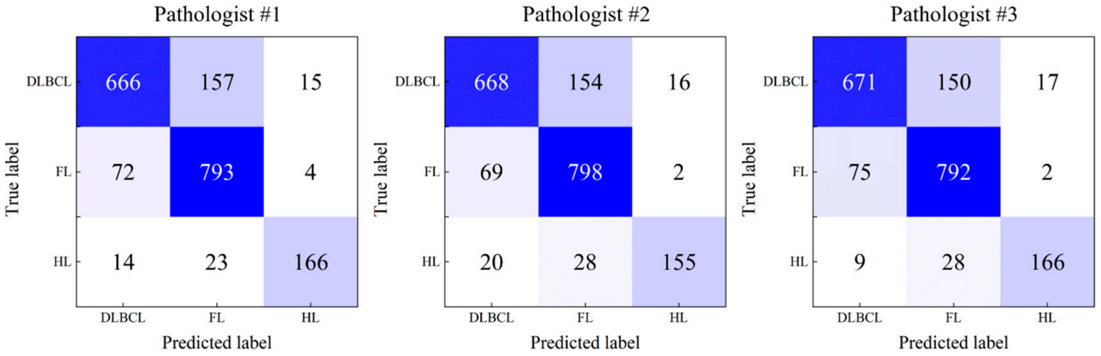

Supplementary Figure S1. The confusion matrix of the diagnostic results after three hematopathologists independently diagnosed all WSIs in the TCH dataset. The lymphoma subtypes of all slides have been determined by immunohistochemistry or genetic testing and are regarded as the true labels. Before the diagnosis, the three hematopathologists already knew that the subtype of the lymphoma slides should be one of DLBCL, FL, and HL.

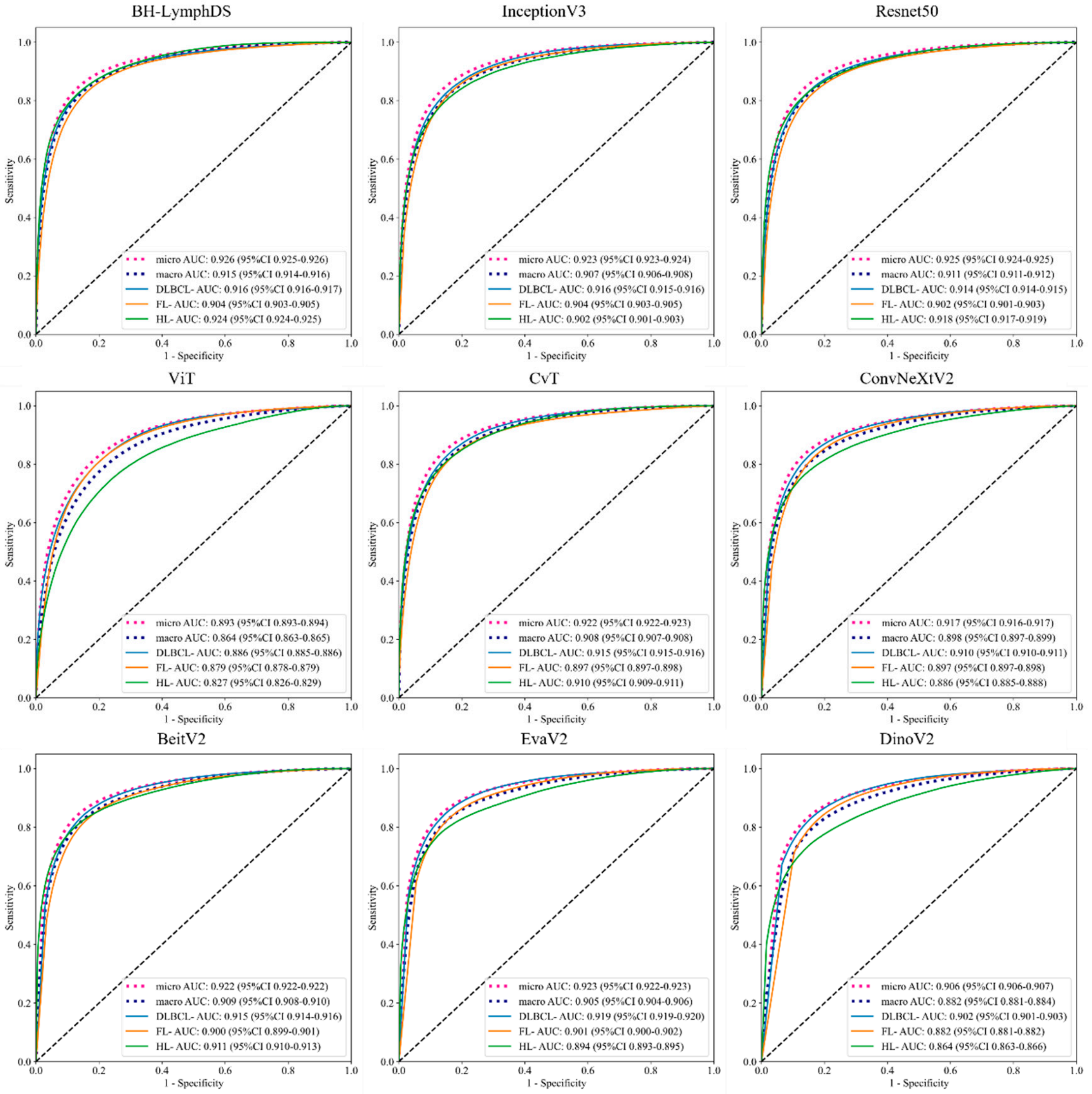

Supplementary Figure S2. The ROC curves and AUC values for predicting lymphoma subtype of patch level images in the internal testing cohort when using various backbone models. Proposed BH-LymphDS takes Swin Transform as the backbone model. Other backbone models include InceptionV3, Resnet50, Vision Transform, Convolutional Vision Transformer, ConvNeXtV2, BeitV2, EvaV2 and DinoV2.

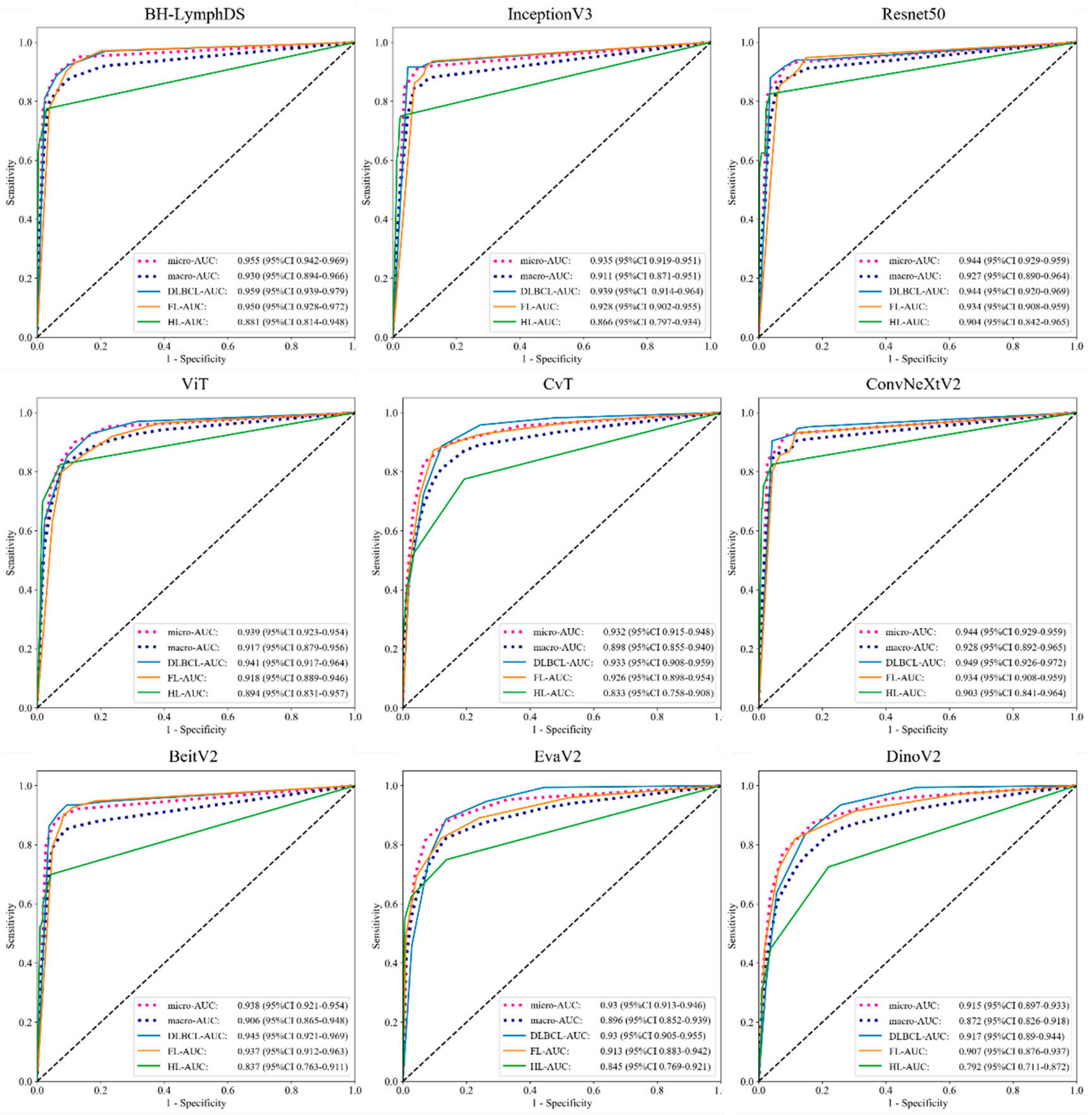

Supplementary Figure S3. The ROC curves and AUC values for predicting lymphoma subtype of each WSI in the internal testing cohort when using various backbone models.

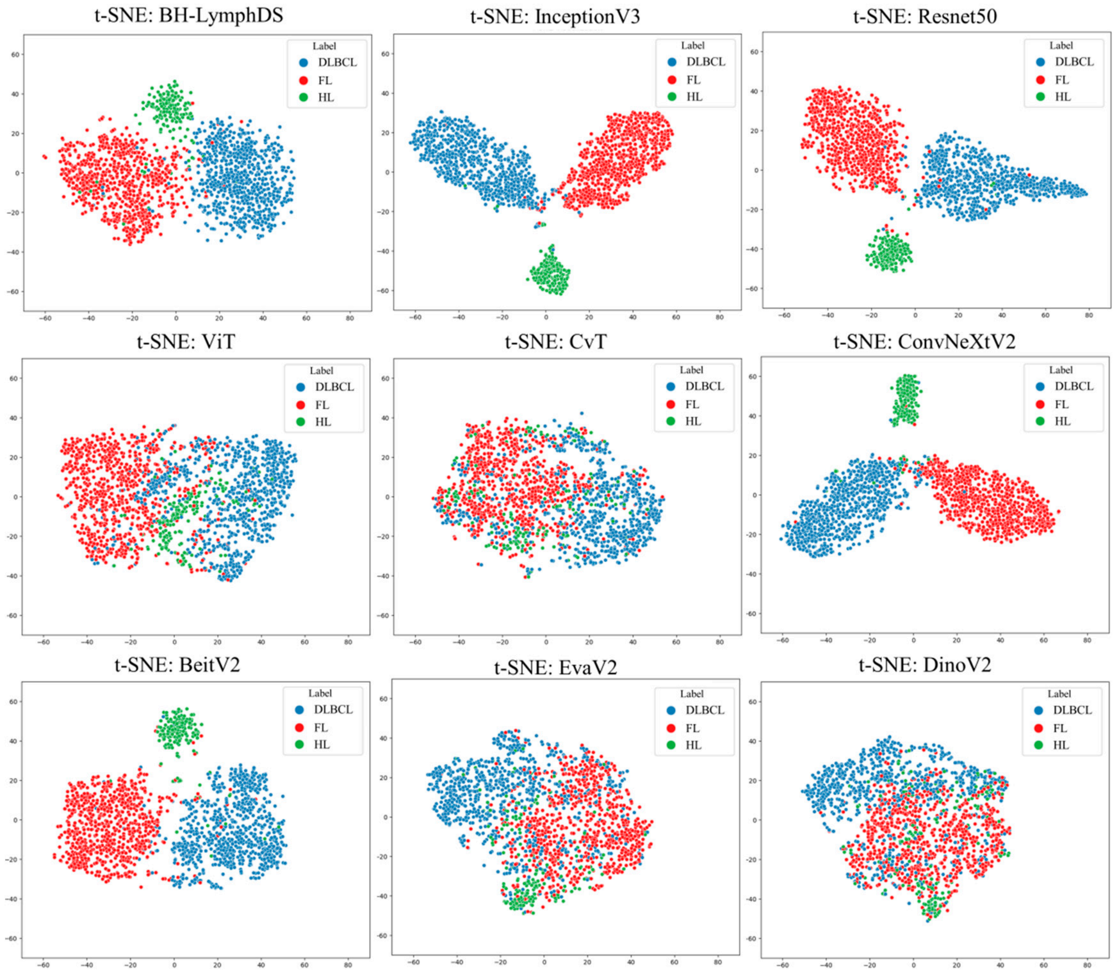

Supplementary Figure S4. The two-dimensional t-SNE projection of the WSI level feature space for predicting the internal testing cohort with the use of various backbone models.

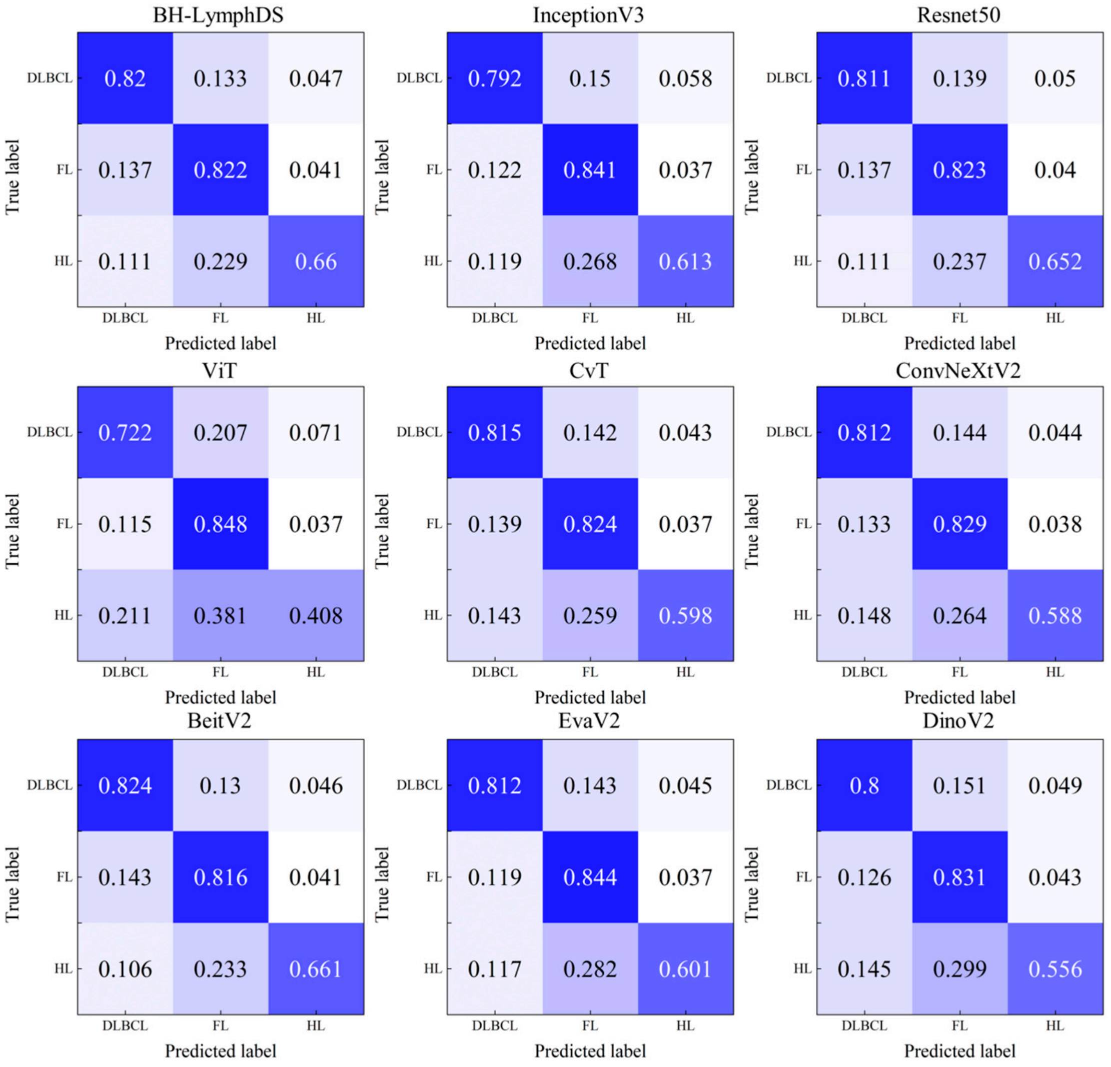

Supplementary Figure S5. The patch level confusion matrix of various backbone models. The normalization was carried out on each row of all the confusion matrices.

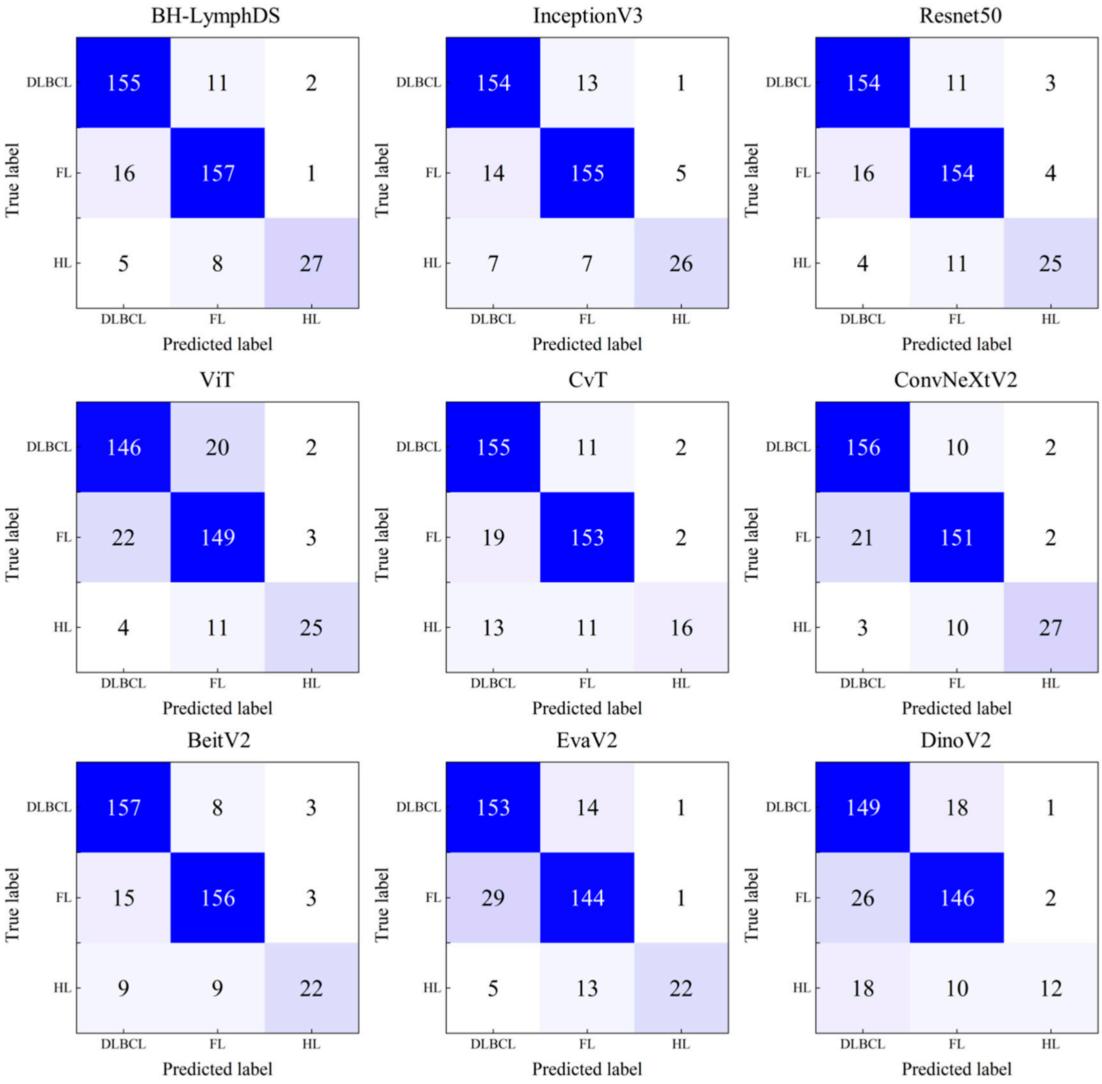

Supplementary Figure S6. The WSI level confusion matrix of various backbone model.

Supplementary Table S1. WSI level system performance of various backbone models.

Task	Model Backbone	Accuracy	AUC	95% CI	Sensitivity	Specificity	Precision	F1 Score
DLBCL	BH-LymphDS	0.911	0.959	0.939-0.979	0.923	0.902	0.881	0.901
	InceptionV3	0.908	0.939	0.914-0.964	0.917	0.902	0.880	0.898
	Resnet50	0.911	0.944	0.92-0.969	0.917	0.907	0.885	0.901
	ViT	0.874	0.941	0.917-0.964	0.869	0.879	0.849	0.859
	CvT	0.882	0.933	0.908-0.959	0.923	0.850	0.829	0.873
	ConvNeXtV2	0.906	0.949	0.926-0.972	0.929	0.888	0.867	0.897
	BeitV2	0.908	0.945	0.921-0.969	0.935	0.888	0.867	0.900
	EvaV2	0.872	0.930	0.905-0.955	0.911	0.841	0.818	0.862
	DinoV2	0.835	0.917	0.89-0.944	0.887	0.794	0.772	0.825
FL	BH-LymphDS	0.906	0.950	0.928-0.972	0.902	0.909	0.892	0.897
	InceptionV3	0.898	0.928	0.902-0.955	0.891	0.904	0.886	0.888
	Resnet50	0.890	0.934	0.908-0.959	0.885	0.894	0.875	0.880
	ViT	0.853	0.918	0.889-0.946	0.856	0.851	0.828	0.842
	CvT	0.887	0.926	0.898-0.954	0.879	0.894	0.874	0.877
	ConvNeXtV2	0.887	0.934	0.908-0.959	0.868	0.904	0.883	0.875
	BeitV2	0.908	0.937	0.912-0.963	0.897	0.918	0.902	0.899
	EvaV2	0.851	0.913	0.883-0.942	0.828	0.870	0.842	0.835
	DinoV2	0.853	0.907	0.876-0.937	0.839	0.865	0.839	0.839
HL	BH-LymphDS	0.958	0.881	0.814-0.948	0.675	0.991	0.900	0.771
	InceptionV3	0.948	0.866	0.797-0.934	0.650	0.982	0.813	0.722
	Resnet50	0.942	0.904	0.842-0.965	0.625	0.980	0.781	0.694
	ViT	0.948	0.894	0.831-0.957	0.625	0.985	0.833	0.714
	CvT	0.927	0.833	0.758-0.908	0.400	0.988	0.800	0.533
	ConvNeXtV2	0.955	0.903	0.841-0.964	0.675	0.988	0.871	0.761
	BeitV2	0.937	0.837	0.763-0.911	0.550	0.982	0.786	0.647
	EvaV2	0.948	0.845	0.769-0.921	0.550	0.994	0.917	0.688
	DinoV2	0.919	0.792	0.711-0.872	0.300	0.991	0.800	0.536

Supplementary Table S2. Hyperparameters of various backbone models.

Model Backbone	BH-LymphDS (SwinTransformer)	ViT	CvT	ConvNeXtV2	BeitV2	EvaV2	DinoV2
Layers	24	12	13	36	12	12	12
Heads	[4, 8, 16, 32]	12	[1, 3, 6]	-	12	12	12
Patch Size	4	32	[7, 3, 3]	4	16	14	14
Head Activation	GELU	GELU	GELU	GELU	GELU	GELU	GELU
Embedding Dimension	128	768	[64, 192, 384]	[128, 256, 512, 1024]	768	768	768
Drop Path Rate	0.1	0.1	0.1	0.1	0.1	0.1	0.1
Normalize Last Layer	True	True	True	True	True	True	True
Optimizer	SGD	SGD	SGD	SGD	SGD	SGD	SGD
Batch Size	14	64	64	64	64	64	64
Learning Rate	0.01	0.01	0.01	0.01	0.01	0.01	0.01
Max Epochs	30	30	30	30	30	30	30
Learning Rate Schedule	Cosine	Cosine	Cosine	Cosine	Cosine	Cosine	Cosine
Feature Dimension	1024	768	384	1024	768	768	768
Other Parameters	Window Size=7	-	-	Kernel Size=7	-	-	-

Supplementary Table S3. Anatomical distribution of WSIs in the TCH cohort

Characteristic		No. of WSIs
Anatomical biopsy site		
	Head and Neck Region	443
	Axillary and Upper Extremity	287
	Inguinal and Lower Extremity	221
	Thoracic Region	158
	Abdominopelvic Region	443
	Gastrointestinal Tract	358
Nodal and Extranodal Lymphoma		
	Nodal lymphoma	1323
	Extranodal lymphoma	587
